# *Dnmt3b* knock-down in enteric precursors reveals a possible mechanism by which this *de novo* methyltransferase is involved in the enteric nervous system development and the onset of Hirschsprung disease

**DOI:** 10.18632/oncotarget.22473

**Published:** 2017-11-16

**Authors:** Ana Torroglosa, Leticia Villalba-Benito, Raquel María Fernández, María José Moya-Jiménez, Guillermo Antiñolo, Salud Borrego

**Affiliations:** ^1^ Department of Genetics, Reproduction and Fetal Medicine, Institute of Biomedicine of Seville, University Hospital Virgen del Rocío, CSIC, University of Seville, Seville 41013, Spain; ^2^ Center for Biomedical Network Research on Rare Diseases, Seville 41013, Spain; ^3^ Department of Pediatric Surgery, University Hospital Virgen del Rocío, Seville 41013, Spain

**Keywords:** Hirschsprung disease, DNMT3b, ENS development, P53, P21

## Abstract

Hirschsprung disease (HSCR, OMIM 142623) is a pathology that shows a lack of enteric ganglia along of the distal gastrointestinal tract. This aganglionosis is attributed to an abnormal proliferation, migration, differentiation and/or survival of enteric precursor cells (EPCs) derived from neural crest cells (NCCs) during the enteric nervous system (ENS) embryogenesis. DNMT3b *de novo* methyltransferase is associated with NCCs development and has been shown to be implicated in ENS formation as well as in HSCR. In this study we have aimed to elucidate the specific mechanism underlying the DNMT3b role in such processes. We have performed the knockdown of *Dnmt3b* expression (*Dnmt3b*-KD) in enteric precursor cells (EPCs) to clarify its role on these cells *in vitro*. Moreover, we have analyzed several signaling pathways to determine the mechanisms responsible for the effect caused by *Dnmt3b*-KD in EPCs. Our results seem to support that *Dnmt3b*-KD promotes an increase EPCs proliferation that may be mediated by P53 and P21 activity, since both proteins were observed to be down-regulated in our *Dnmt3b*-KD cultures. Moreover, we observed a down-regulation of *P53* and *P21* in HSCR patients. These results lead us to propose that DNMT3b could be involved in HSCR through P53 and P21 activity.

## INTRODUCTION

Hirschsprung disease (HSCR, OMIM 142623), the most common neurocristopathy in humans (1:5000 newborns), is characterized by the absence of enteric ganglia along variable lengths of the distal gastrointestinal tract, resulting in severe intestinal dysfunction [1]. Based on the length of the aganglionic region, HSCR phenotypes are classified as: short-segment forms (S-HSCR) which include patients with aganglionosis as far as the splenic flexure, long-segment forms (L-HSCR) in which aganglionosis extends beyond the splenic flexure and total colonic aganglionosis forms (TCA) [2]. Such aganglionosis is attributed to a failure of the proliferation, migration, differentiation and/or survival of the enteric precursors cells (EPCs) derived from NCCs during embryonic development of Enteric Nervous System (ENS).

The ENS formation is a complex process that requires a specific gene expression pattern at each stage, and alterations throughout such pattern can lead to drastic consequences, as evidenced by the aganglionosis observed in HSCR [3]. There are several mechanisms that regulate gene expression during the embryogenesis of ENS. Among them, epigenetic mechanisms are acquiring increasing evidence to play a major role in the onset of this disease [4]. One well characterized epigenetic mechanism regulating gene expression is DNA methylation, which in mammalian is mediated by three DNA methyltransferases: DNMT1, DNMT3a and DNMT3b [5]. DNMT1 is the most abundant DNA methyltransferase in mammal cells, and is responsible for the maintenance of DNA methylation. DNMT3a and DNMT3b function as *de novo* methyltransferases and together are responsible for methylation pattern acquisition during gametogenesis, embryogenesis, and somatic tissue development as well as for maintaining the silence of transposable elements and enhancing the stability of genome. [6, 7]

DNMT3a and DNMT3b have been shown to be essential for the normal NCCs development in mammals and both of them play important roles in diseases related to this process [8–12]. Neural crest malformation can lead to craniofacial defects like cleft lip palate, heart septation defects and aganglionosis of the colon [13–15]. Specifically the *Dnmt3b* knockout mouse embryo shows rostral neural tube defects and growth impairment [8]. In human embryonic stem cells, knockdown of *DNMT3b* accelerates neural crest differentiation and increases the expression of neural crest specifier genes (*PAX3, PAX7, FOX3, SOX10 and SNAIL2*) [12]. Moreover, *DNMT3b* shows a decrease of expression in enteric precursors cells (EPCs) from HSCR patients compared with controls, and this result translates into a lower level of DNA methylation in HSCR patients [16].

Although it has been demonstrated the implication of *DNMT3b* in the ENS formation as well as in HSCR, its role in this process is unknown. Therefore, we have performed the study of the *Dnmt3b*-KD in mouse EPCs that were grown as Neurosphere like-bodies (NLBs). In these NLBs cultures we have evaluated the effect of *Dnmt3b*-KD on cell proliferation, differentiation and survival. In addition, we have analyzed several signaling pathways to determine the possible mechanisms responsible for the effect caused by *Dnmt3b*-KD in EPCs.

## RESULTS

### *Dnmt3b-*KD in EPCs resulted in an increase of proliferation and/or survival

**Figure 1:**
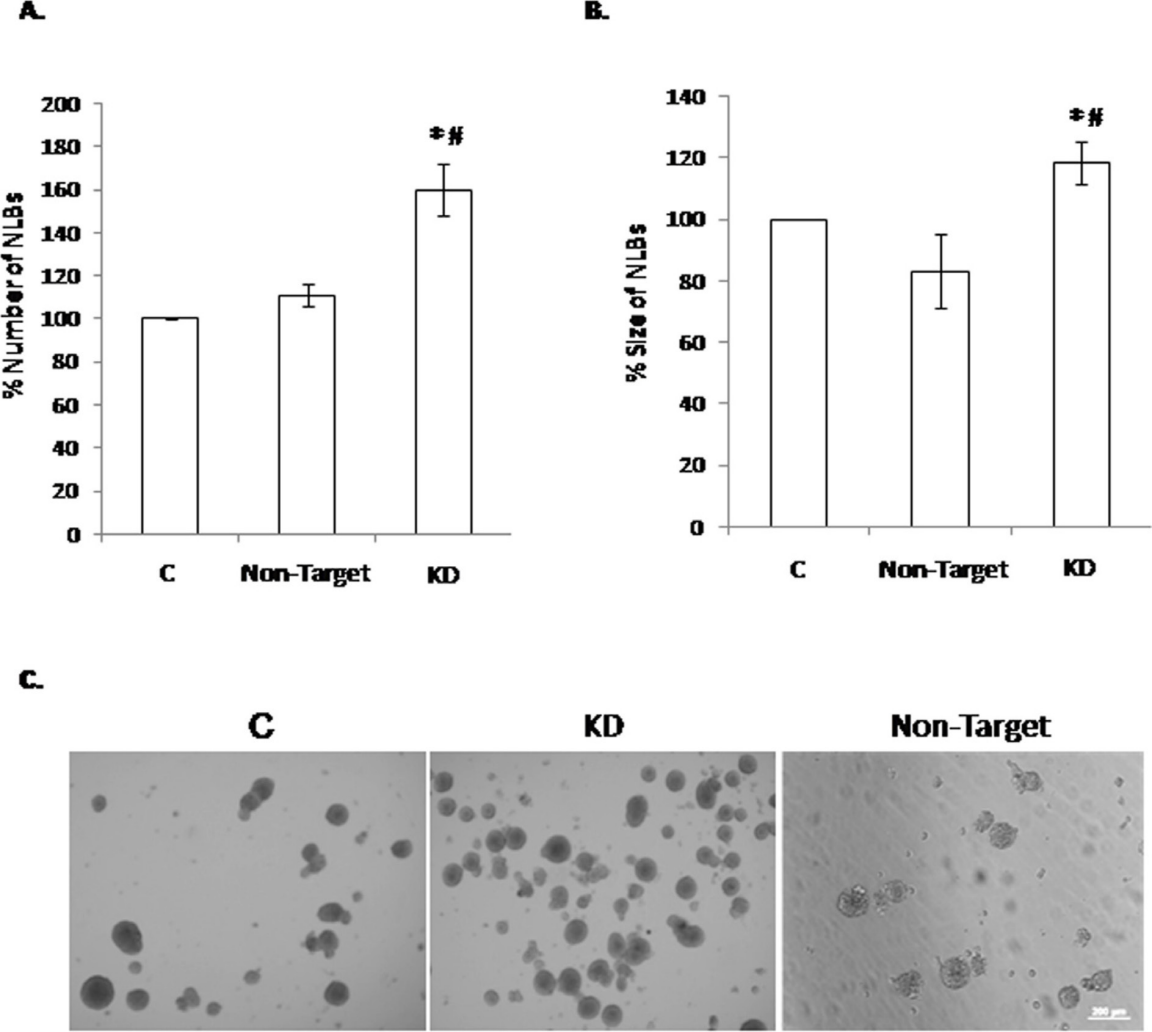
Effect of the *Dnmt3b*-KD on the NLBs cultures (**A**) Effect of *Dnmt3b-*KD in the number of NLBs (*p* value KD/C = 0.0004, KD/Non-Target = 0.02). (**B**) Effect of *Dnmt3b-*KD in the size of NLBs. (**C**) Representative image showing the appearance of each one of the three different culture conditions (*p* value KD/C = 0.02, KD/Non-Target = 0.03). Scale bar 200 µm. Data are represented as mean ± SEM.

First we evaluated the expression of *Dnmt3b* in the NLBs cultures by SYBR green real-time RT-PCR technique. We could observe that this gene is expressed in EPCs from NLBs cultures (Supplementary Table 1). The *Dnmt3b*-shRNA that produced higher reduction in the gene expression level was *Dnmt3b*-shRNA1 (KD) (Supplementary Figure 1). Then, we analyzed the possible effect of *Dnmt3b*-KD in the NLBs cultures and observed a significant increase of NLBs number in KD compared with the control (C) and shRNA Non-Target Control (Non-Target) conditions (Figure 1A). We also detected an increase in NLBs size in the KD group compared with C and Non-Target groups. However Non-Target showed a decrease of NLBs size in comparison with the C group (Figure 1B). Figure 1C shows representative images of NLBS cultures under the three different conditions.

### *Dnmt3b*-KD effect on EPCs phenotypes

**Figure 2:**
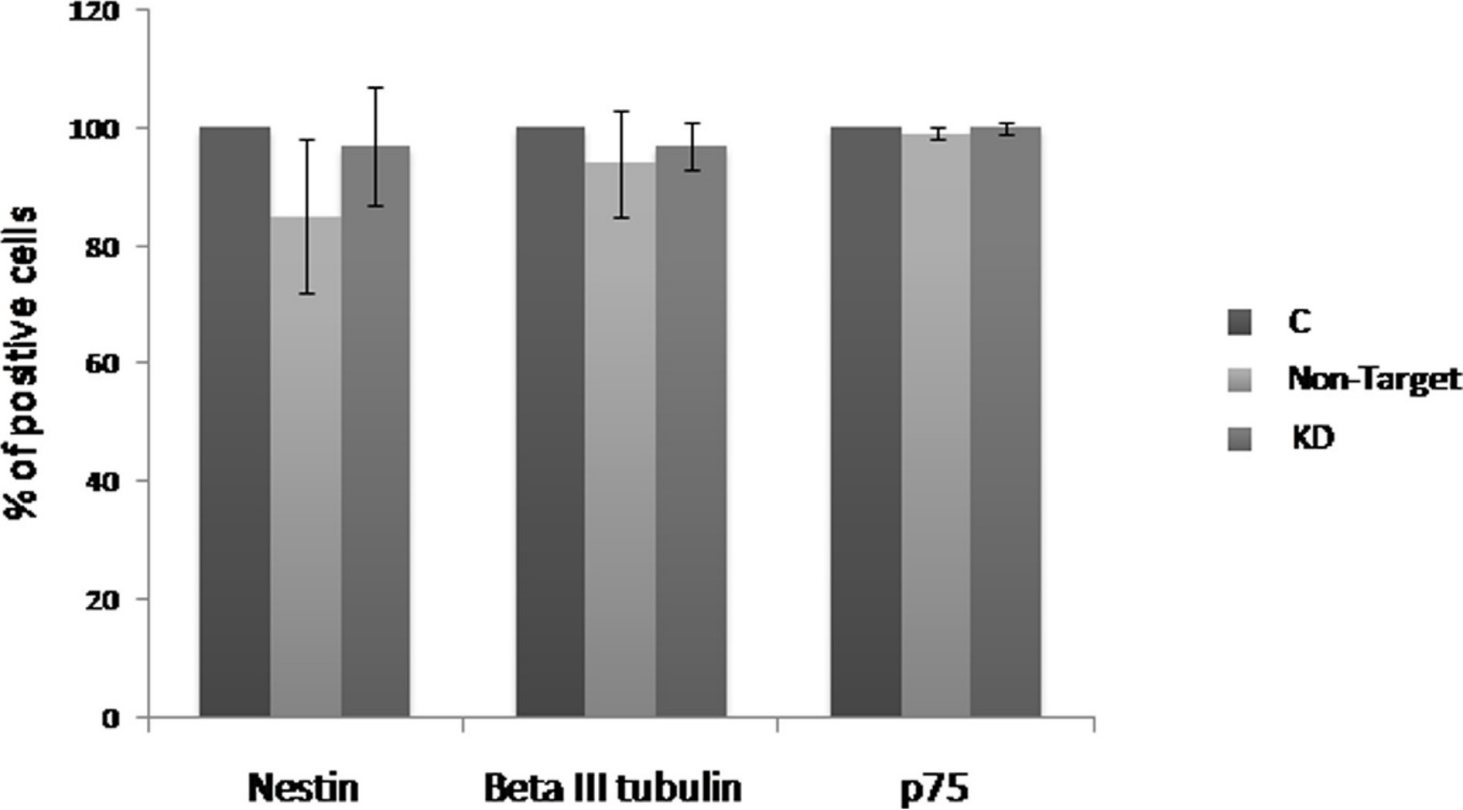
Effect of the *Dnmt3b*-KD on the cell phenotypes derived from NLBs Effects of *Dnmt3b*-KD on the EPCs that express Nestin (Nest), β-III tubulin (β-III), or p75 markers in the different culture conditions. Data are represented as mean ± SEM.

Evaluation of the *Dnmt3b*-KD effect on the neurogenesis was performed by flow cytometry in the NLBs cultures. With this purpose we analyzed the p75, Nestin and βlll-Tubulin markers, and observed that Non-Target and KD cultures showed a decrease of Nestin+ (15% and 3% respectively) and βlll-Tubulin+ cells (6% and 3% respectively) compared to C culture. On the other hand, p75 expression level was not different among the groups (Figure 2). The decrease detected in the number of Nestin + and βlll-Tubulin+ cells was due to the treatment with shRNA and we observed that the *Dnmt3b*-KD seems to counteract this effect resulting in an increase of these cell types in KD compared with Non-Target cultures. We also analyzed the double positive cells for the studied markers. Nestin+/p75+, βlll-Tubulin+/p75 and Nestin/βlll-Tubulin+ combinations were analyzed (Supplementary Figure 2). It is worthy to note that there were no statictically significant changes in cell phenotypes within the *Dnmt3b*-KD cultures.

Identification of the mechanisms implicated in cell proliferation and/or survival regulated by DNMT3b in EPCs

### *Dnmt3b*-KD effect on the MAPK/ERK, PI3K/AKT and STAT3 pathways activation

**Figure 3:**
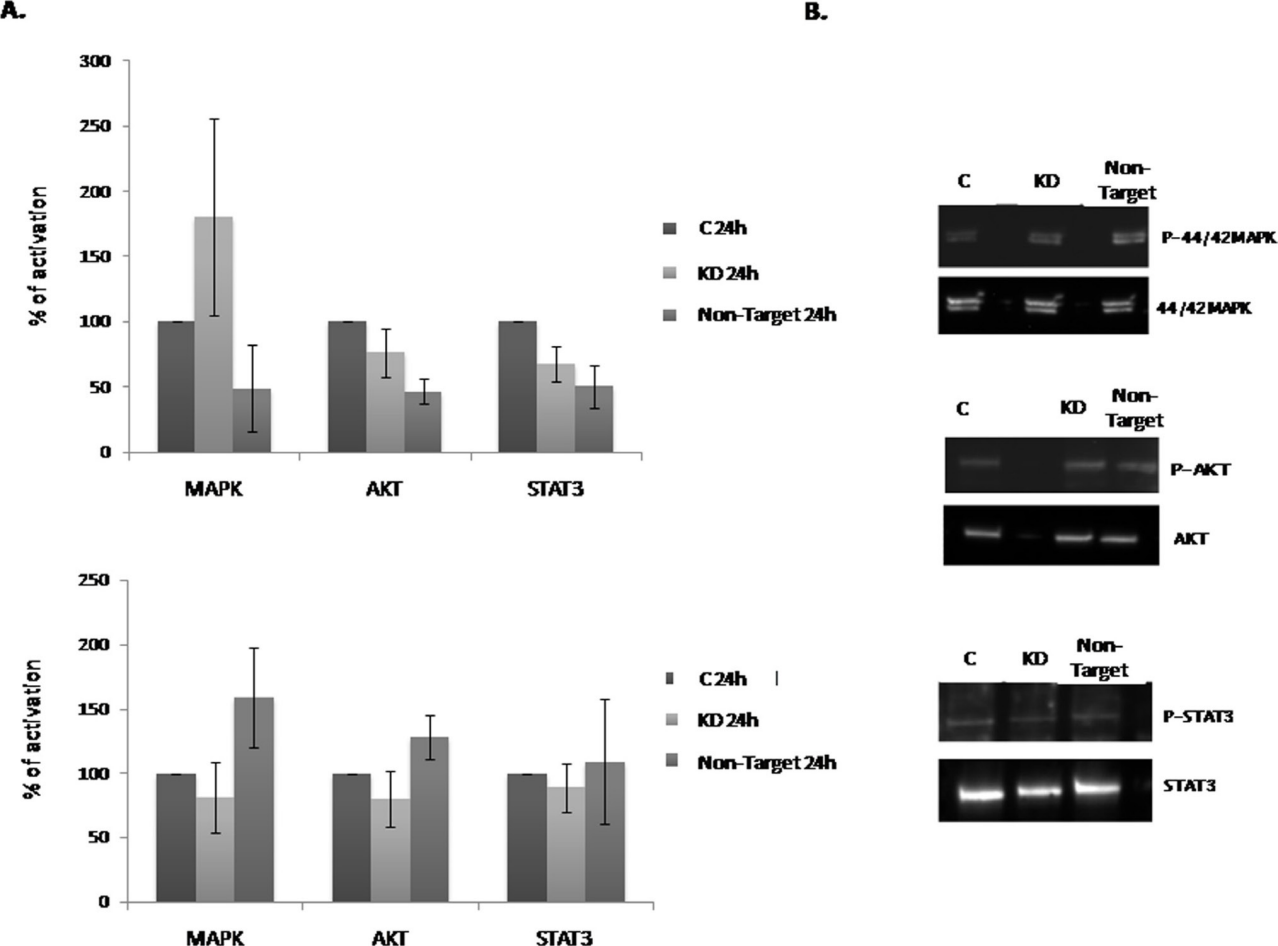
Effect of the *Dnmt3b*-KD on the cell signaling pathways activation in NLBs cultures (**A** and **B**) Graphs showing the effect of *Dnmt3b-*KD in the different culture conditions on the activation of MAPK/ERK (MAPK), PI3K/AKT (AKT) and STAT3 pathways at 24 h and 7 div respectively. Data are represented as mean ± SEM.

The observation of the increase in the NLBs number and size in *Dnmt3b*-KD cultures, prompted us to investigate the signaling pathways that may be involved in this effect. For this purpose we studied MAPK/ERK, PI3K/AKT and STAT3 activation. All these pathways have been related to cell proliferation and survival. We analyzed the pathways activation at 24 h and observed that MAPK/ERK pathway in KD cultures was more activated than in the C and Non-Target groups (Figure 3A). On the other hand, both PI3K/AKT and STAT3 activations were subtly lower in KD and Non-Target than in the C cultures. At 7 days *in vitro* (7 div.) we observed that all pathways showed the same level of activation in all the culture conditions (Figure 3B). In summary, we detected weak modifications in the activation of these pathways in *Dnmt3b*-KD compared with C cultures. Therefore these signaling pathways do not seem related to the effect on EPCs proliferation and/or survival that the *Dnmt3b*-KD cultures showed.

### *Dnmt3b*-KD effect on *Bcl-xL* and *p53* expression

**Figure 4:**
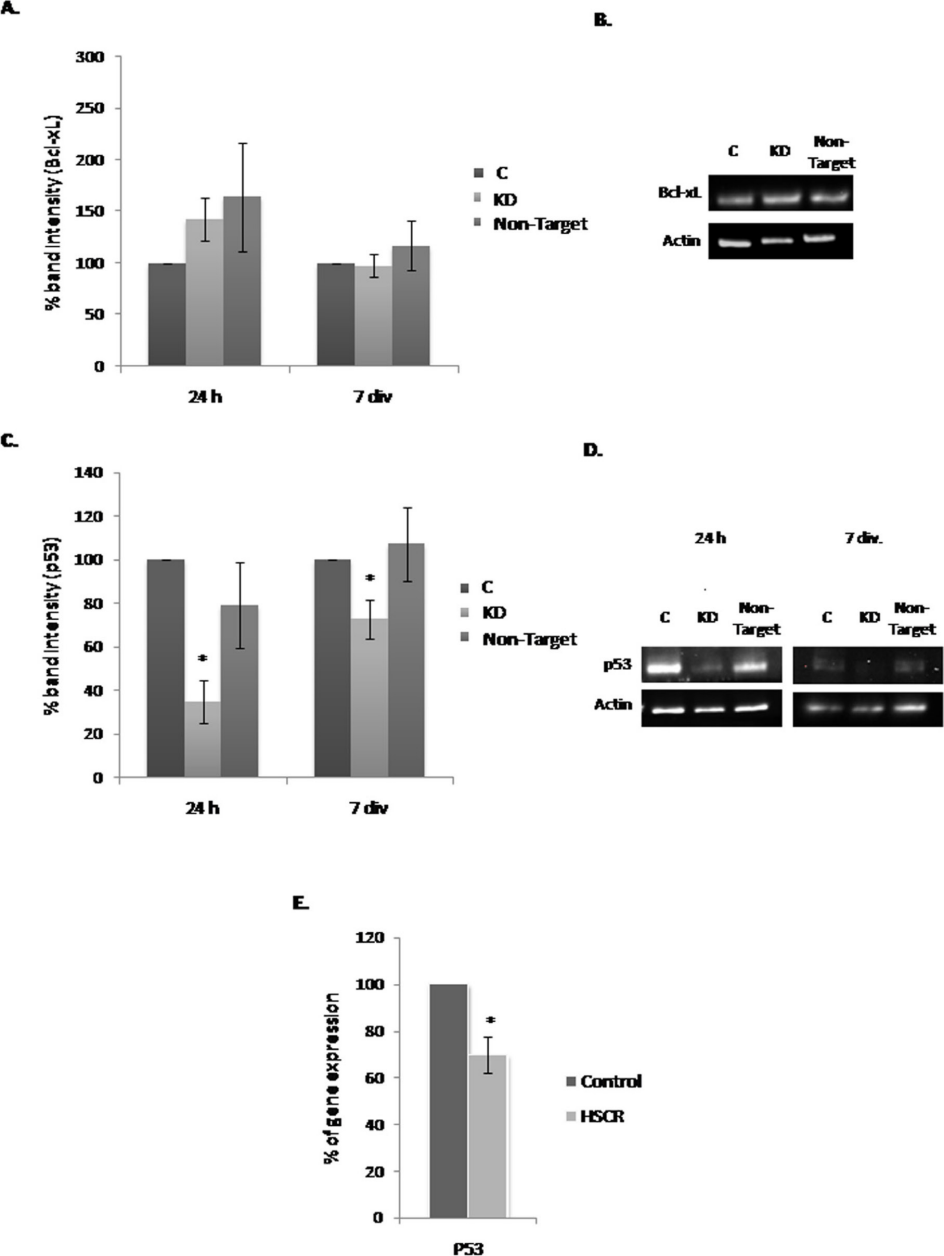
Effect of the *Dnmt3b*-KD on the expression of *Bcl-xL* and *p53* in NLBs cultures (**A**) Effect of *Dnmt3b*-KD on the *Bcl-xL* expression in the three different conditions cultures at 24 h and 7 div. (**B**) Representative image showing the effect observed in the *Bcl-xL* expression level in the three different conditions at 24 h and 7 div. (**C**) Effect of *Dnmt3b*-KD on the *p53* expression in the three different conditions cultures at 24 h and 7 div. (*p* value KD/C 24 h = 0.0006, KD/C 7 div. = 0.028). (**D**) Representative image showing the effect observed in the *p53* expression level in the three different conditions at 24 h and 7 div. Actin was used as a loading control. (**E**) Graph showing the difference between *P53* expression levels in EPCs from HSCR patients with *DNMT3b* low expression and in EPCs from controls (*p* value = 0.0003). Data are represented as mean ± SEM.

*Bcl-xL* and *p53* expression levels were also analyzed given that both proteins have been widely associated with cell survival. In the case of *BcL-xL*, no significant changes in its expression level were identified when we compared all the culture conditions (Figure 4A and 4B). In contrast, *p53* at 24 h and 7 div. showed a decrease in its expression level in KD compared with the C and Non-Target cultures, being this difference more pronounced at 24 h of infection (Figure 4C). Figure 4D corresponds to a representative immunoblot assay image showing the decrease of P53 in *Dnmt3b*-KD cultures. Based in this result, we analyzed the *P53* expression level in EPCs from HSCR patients compared to controls. We observed that the EPCs from HSCR patients with a *DNMT3b* low expression have a significant decrease in the *P53* expression (30.3%) compared to controls, Figure 4E.

### DNMT3b seems to regulate the cell cycle through the P53/P21 activity

**Figure 5:**
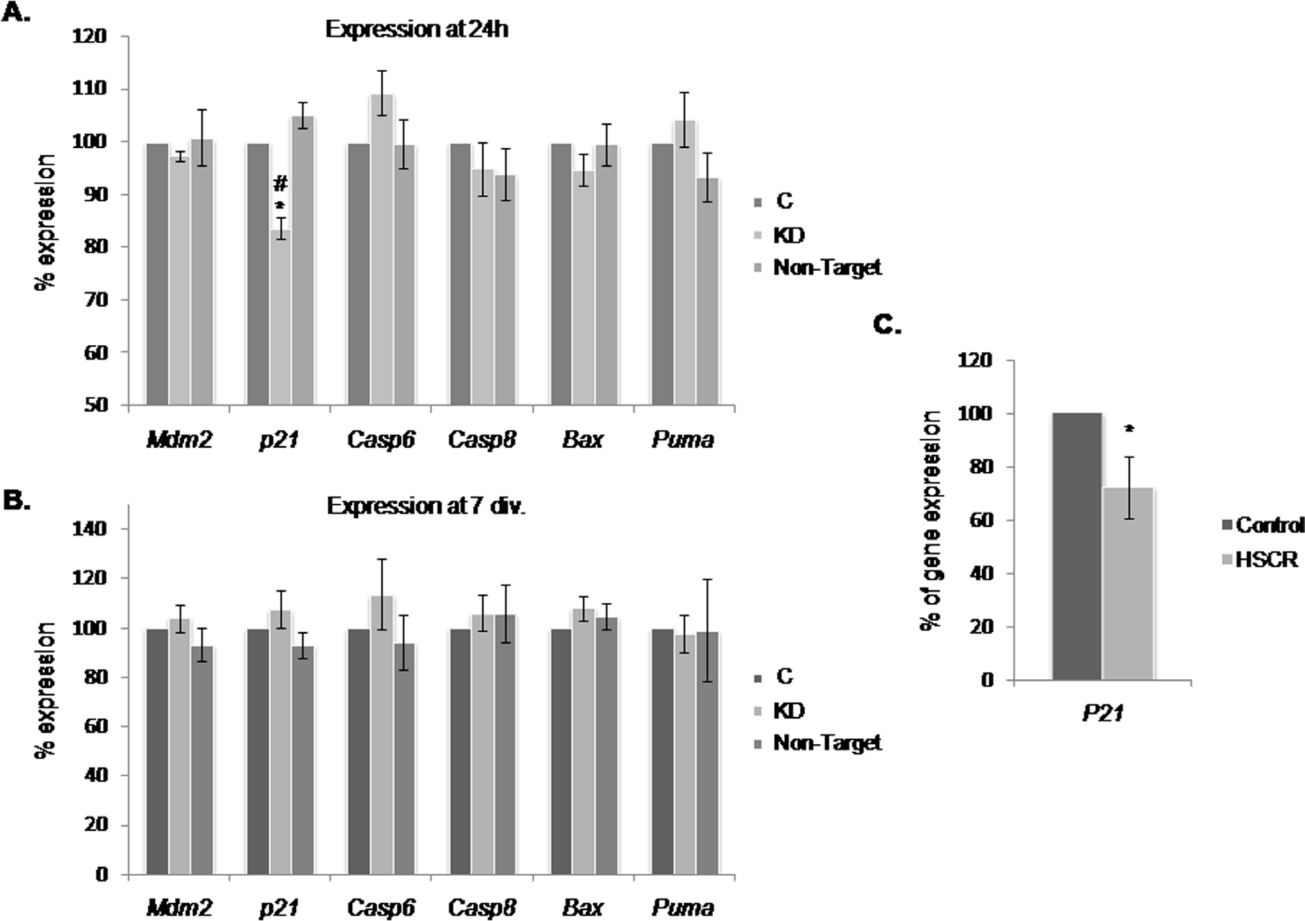
Effect of the *Dnmt3b*-KD on P53-pathways related genes in NLBs cultures (**A** and **B**) Graph showing the expression levels of *Mdm2*, *p21*, *Casp6*, *Casp8*, *Bax* and *Puma* in *Dnmt3b*-KD EPCs cultures at 24 h and 7 div. A significant decrease of expression of *p21* was detected at 24 h (*p* value KD/C = 0.002, KD/Non-Target = 0.001). (**C**) Graph showing the difference between *P21* expression levels in EPCs from HSCR patients with *DNMT3b* low expression and in EPCs from controls (*p* value = 0.02). Data are represented as mean ± SEM.

Our results suggested that the increase in the cell growth observed in *Dnmt3b*-KD cultures is mediated by *p53* down-regulation. This prompted us to evaluate the expression level at 24 h and 7 div. of a group of genes related with P53 that participate in pathways either implicated in proliferation (*cyclin-dependent kinase inhibitor p21*) or in apoptosis (*Bax*, *Puma*, *Casp6* and *Casp8*). We also wanted to know if the inhibitor of *p53* expression, *Mdm2,* was responsible of the *p53* down-regulation observed in *Dnmt3b*-KD. As a result, we just observed a decrease in the expression level of *p21* (17%) at 24 h (Figure 5A), while at 7 div., no significant changes were detected in any of the analyzed genes (Figure 5B). Therefore, we decided to analyze the expression level of *P21* in EPCs from those HSCR patients with a low expression of *DNMT3b* in comparison with that of controls, and observed a *P21* down-regulation (27 %) in EPCs from HSCR patients (Figure 5C).

## DISCUSSION

In this work we have studied *Dnmt3b*-KD in mouse EPCs cultures in order to further investigate the role of this methyltranferase on EPCs, given its significant down-regulation observed in HSCR patients [16].

We noticed a significant increase of NLBs number and size, which suggests the possible regulatory role of *Dnmt3b* in the proliferation and/or survival on EPCs. In this sense, DNMT3b has been widely related with these cellular processes and specifically its depletion in different human cells has shown to produce extremely variable effects [17–19]. There are many studies that have demonstrated that a reduction of DNMT3b protein levels induces antiproliferative effects in human cancer cells which were attributed to the demethylation and reactivation of tumor suppressor genes. Therefore, *de novo* methyltransferase DNMT3b is implicated in the establishment of gene-specific hypermethylation during cancer development and progression [17–20]. Recently, it has been also described such DNMT3b antiproliferative effect on vascular smooth muscle cells from human [21]. Conversely, Umehara *et al.* showed that DNMT3b induces the initiation of stem/progenitor cell differentiation via down-regulation of embryonic stem cell proliferation coordinated with the down-regulation of *Oct3/4* and *Nanog* genes [22]. Therefore, this study suggests that DNMT3b plays an important role in the initial process of stem cell differentiation and in the inhibition of their proliferation. Such role of DNMT3b is in accordance with the results obtained in *Dnmt3b-*KD NLBs cultures, where the DNMT3b depletion showed an increase of the EPCs proliferation and/or survival.

On the other hand, cell differentiation status is defined by the gene expression profile, which is coordinately controlled by epigenetic mechanisms. There are *in vivo* and *in vitro* studies that suggest that DNMT3b is required for the initial steps of progenitor cell differentiation, and DNMT3a is required for maturation processes of cell differentiation [23, 24]. In our case, the EPCs phenotypes in *Dnmt3b*-KD cultures did not show significant changes when they were compared with the C condition. A decrease in the percentage of Nestin+ (p75+ or βlll-Tubulin+) and βlll-Tubulin+ (p75+ or Nestin+) cells was noticed, though in Non-Target cultures the decrease was more pronounced. Therefore, we did not observe that *Dnmt3b-*KD triggered a loss of differentiation capacity in our NLBs culture, in contrast with which has been previously described [23–25].

Taking to account our whole results, we propose that DNMT3b could have an antiproliferative effect on EPCs and it does not affect their neurogenic capacity. In order to know the mechanisms by which DNMT3b act on EPCs proliferation and/or survival, we studied MAPK/ERK, PI3K/AKT and STAT3 pathways activation in the NLBs cultures. We selected such pathways because it has been shown that RET the main protein implicated in the onset of HSCR [26] can activate them [27–29]. In addition such pathways have a role on proliferation, survival, apoptosis, migration, and differentiation of EPCs and they are most likely to be affected by HSCR-related changes [30]. We did not observe significant changes in these signaling pathways in KD in comparison with C cultures. However, when we analyzed the *p53* and *Bcl-xL* expression, both proteins implicated in the apoptosis and/or cell cycle regulation, we observed that *p53* showed a significant lower expression maintained in time in KD cultures and *Bcl-xL* did not show significant changes. We noticed that this decrease of *p53* expression does not seem to be regulated by MDM2 in EPCs from mouse in response to the *DNMT3b* lower expression. Based on these results, we evaluated the expression level of *P53* in EPCs from HSCR patients that had previously shown a decline in *DNMT3b* expression [16], and a *P53* down-regulation was also detected. P53 is a well-known protein involved in the induction of apoptosis [31, 32]. Recently, it has been published the relationship of P53 with the NCCs development and with the onset of neurocristopathies in zebrafish [33]. On the other hand P53 also has been related with DNMT3b, since it has been identified that the hypermethylation of *p53* promoter by DNMT3b causes a decrease of its expression level and promotes an increase of cell proliferation [21]. In concordance with this effect on cell growth, the decrease observed in *p53* expression level in our EPCS (KD*-Dnmt3b* and HSCR patients) correlated with the *p21* down-regulation. The cyclin-dependent kinase inhibitor *p21* is a P53 target gene known to play a key role in P53-mediated cell cycle arrest [34, 35]. Postnatal P53-deficient mice exhibit increased proliferation within the Subventricular Zone and increased neurogenesis [36]. Furthermore, the neural precursor cells derived from P53-null mice exhibit reduced apoptosis and enhanced proliferation [37]. Taking into account all of these correlations, we propose a possible antiproliferative effect for DNMT3b through P53/P21 activity.

Nervous System development is a process that integrates cell proliferation, cell differentiation cell cycle arrest and programmed cell death. Apoptosis is a conserved mechanism as well as a fundamental developmental process by which the final cell number in the nervous system is established [38]. It is worthy to note that Bordeaux *et al.* described a pro-apoptotic role of RET in ENS development by inducing cell death in the NCCs that migrate to areas beyond ligands availability [39]. In addition it has been described that cell cycle arrest plays an important role in the establishment of the critical cell population for the correct formation of the Nervous System [40]. Therefore cell death and cell cycle arrest seem to be important processes required during early development of the ENS. This reinforces the idea that control of NCCs number in the developing gut is critical for normal ENS formation [41]. In this sense, our results would correlate with the role that the cell cycle arrest plays in NCCs development, and this regulation apparently seems to occur through P53 and P21 activity.

In conclusion, this study suggests that DNMT3b has an antiproliferative effect on mouse EPCs that could to be mediated through the P53 and P21 activity. This proposed mechanism is supported by the observed correlation between the down-regulation of *P53* and *P21* in EPCs from HSCR patients that had previously shown a lower expression of *DNMT3b*. Therefore, *DNMT3b* down-regulation is concomitant with a decrease in the *P53* and *P21* expression levels and with an increase of EPCs proliferation, concluding that all these events together might contribute to an anomalous formation of the ENS and, ultimately, to the onset of HSCR.

## MATERIALS AND METHODS

### Gut samples from mice and human

Gut tissue was obtained from CD1 mice (P7) and from human postnatal tissues of ganglionic gut from 3 sporadic non-related patients diagnosed of S-HSCR (male: female = 1:2), as well as from 4 patients with other gastrointestinal disorders (anorectal malformations and enterocolitis) undergoing gut resection surgery at our Hospital that were used as controls (male: female = 2:2). For both HSCR patients and control individuals, age ranged from 3 months to 3 years. All procedures involving mice were performed in accordance with the Guidelines of the European Union Council (86/609/EU) and Spanish regulations (BOE 67/8509-12) for the use of laboratory animals. The written informed consent for surgery, clinical and molecular genetic studies was obtained from all the human participants or their guardians. The study was approved by the Ethics Committee for clinical research of the University Hospital Virgen del Rocío (Seville, Spain) and complies with the tenets of the declaration of Helsinki.

### Culture of enteric precursors cells as NLBs

Gut samples were cut into pieces of 1 mm^2^ and incubated in a solution of 0,26 mg/mL Trypsin Collagenase, 5 mg/mL Dispase, 0,28 mg/mL Hyaluroniodase, 3,3 u/mL Elastase and 0,6 mg/mL Collagenase in phosphate-buffered saline (PBS) for up to 30 minutes at 37°C. Digested tissue was triturated and washed, and the cells were cultured in the following conditions: Dulbecco’s modified Eagle medium (DMEM; 1 mg/mL Glucose) containing 100 U/mL penicillin, 100 g/mL streptomycin, supplemented with 2 mM L-glutamine (Gibco, Life Technology, California, USA), 0.05 mM 2-mercaptoethanol, 1% (v/v) N1 (Sigma Aldrich, Poole, Dorset, UK), 10% (v/v) Human serum, 20 ng/mL basic fibroblast growth factor (bFGF), 20 ng/mL epidermal growth factor (EGF) and 10 ng/mL glial cell derived neurotrophic factor (GDNF) (Peprotech, London, UK). Finally, after 4–7 days *in vitro* (div) the NLBs were formed.

### Gene expression study by quantitative real-time PCR (qRT-PCR)

Purification and synthesis of cDNA from mouse NLBs were performed using the protocol provided by µMACS mRNA isolation Kit and µMACS cDNA Synthesis Kit in a thermo MAKSTM Separator (MACS Miltenyi Biotech, Germany) or RNAeasy Micro kit and RT^2^ First Strand kit (Qiagen, Germany). Expression studies were carried out in an Applied Biosystems 7900HT system (Life Technologies, USA) through SYBR Green method (Bio-Rad, USA). Analysis was performed using the RQ Manager Software (Life Technologies, USA). *Beta*-*Actin or Gapdh* were used as endogenous controls. To determine mRNA levels specific primer pairs for *Dnmt3b*, *P53*, *p21*, *P21*, *Bax*, *Puma*, *Casp6*, *Casp8* and *Mdm2* (Supplementary Table 2) were used. Following the software recommendations, the upper limit of the cycle threshold (Ct) was set to be 35. Therefore we considered positive expression exclusively when Ct values <35.

### *Dnmt3b* shRNA-Expressing Lentiviral Vector and infection of the EPCs

We knocked down the *Dnmt3b* expression in mouse NLBs cultures using lentiviral transduction. We used constructs that constitutively expressed a small hairpin RNA (shRNA) directed against *Dnmt3b* mRNA. As a negative control we employed a shRNA Non-Target Control. Several *Dnmt3b* shRNA-expressing lentiviral vectors were tested (Sigma Aldrich, USA): *Dnmt3b*-shRNA1, *Dnmt3b*-shRNA2, *Dnmt3b*-shRNA3 and *Dnmt3b*-shRNA4 and the multiplicity of infection (MOI) used was 1,5. *Dnmt3b*-shRNA1 was observed to produce a higher reduction in the gene expression level of *Dnmt3b* (Supplementary Figure 1). Therefore, we selected this construction to continue with the approach. The cultures of enteric precursors extracted from mouse gut were divided into 3 groups: uninfected group (C), shRNA Non-Target Control group (Non-Target), and *Dnmt3b*-shRNA1 group (KD). The cultures of single enteric precursors were infected with the shRNAs (KD or Non-Target) and they were maintained during 7 div. in order to obtain NLBs suitable to detect changes in the processes of interest.

### Cell proliferation assays

Enteric precursor cells isolated from mouse gut were grown at a density of 2 × 10^5^ cells/well during 7 div. The number of NLBs/well generated was counted under phase microscopy (IX71, Olympus, Japan) 7 div. later. To measure the NLBs size, images of twenty fields per well were obtained, and the surface of the formed NLBs (at least 60 NLBs per condition) was estimated using the Microimage analysis system software (Olympus, Japan).

### Flow cytometry

The NLBs obtained from the different conditions of culture were enzymatically and mechanically dissociated to obtain a single-cell suspension in which we carried out the cell labeling. The cells were fixed with 4% (weight/volume) paraformaldehyde in 0.1 mol/l phosphate-buffered saline. Then, they were incubated for 1 hour in 2.5% (weight/volume) bovine serum albumin in phosphate-buffered saline and with primary (16 hours at 4°C) and secondary antibodies (1 hour at room temperature). Several primary antibodies were used: anti-Nestin (goat polyclonal; 1:1000) (Santa Cruz Biotechnology, Inc) as a neural precursor cells marker, anti-β III Tubulin (mouse monoclonal; 1:500) as a neuronal precursor cells marker (Merck Millipore, German) and anti-p75 (rabbit polyclonal; 1:500) as a enteric precursor cells marker (Abcam, UK). The secondary antibodies were anti-goat IgG labeled with Cy5, anti-mouse Ig G labeled with Cy2 (1:200; Jackson Immuno Research Laboratories Inc, UK), and anti-rabbit IgG labeled with Alexa Fluor 568 (1:200; Life Technologies, USA) respectively. Flow cytometry analysis data were collected using the LRS II Fortessa cell analyzer (Becton Dickinson, USA). The expressions of Nestin, βIII-Tubulin and p75 markers as well as of different combinations of these markers were analyzed in the EPCs.

### Immunoblot assays

Uninfected, KD and Non-Target NLBs were lysed at 24 h and 7 div. with RIPA Buffer that contained protease and phosphatase inhibitors (Roche, Germany). Supernatants were collected by centrifugation (16000 g) and their protein concentration was measured by the Bradford-based Bio-Rad microassay method. Equal amounts of total protein from each cellular extract (20 mg) were separated by SDS-PAGE and transferred onto a polyvinylidene difluoride membrane. The immunodetection was carried out using the following rabbit polyclonal primary antibodies against the following proteins: extracellular signal-regulated kinase (Erk1/2) (1:1000), pp-Erk (p44/42, Thr202/Tyr204) (1:1000), p-Akt (Ser437) (1:1000), Akt (1:1000), p-Stat3 (Tyr705) (1:1000) and Stat3 (1:1000) for the study of the activation of cell signaling pathways, and p53 (1:1000) and Bcl-xL (1:2000) for the study of cell apoptosis (Cell Signaling Technology, USA). After washing with T-TBS (Tris-buffered salt solution with Tween), the membrane was incubated with the anti-rabbit IgG HRP-linked secondary antibody (1:2000) (Cell Signaling Technology). Bands were made visible by ChemiDoc XRS (Bio-Rad, USA). Quantification was done by photodensitometry with the Image Lab. 3.0 software (Bio-Rad, USA).

### Statistical analysis

Data are presented as the mean ± SEM (Standard Error Mean) of values obtained from at least three assays. Comparisons between values obtained in the C, KD, and Non-Target conditions were analyzed using the Student´s *t*-test. Differences were considered significant when *p* value *<* 0.05.

## SUPPLEMENTARY MATERIALS FIGURES AND TABLES



## Abbreviations

HSCR: Hirschsprung disease
DNMT: DNA methyltransferase
ENS: Enteric Nervous System
EPCs: Enteric Precursor Cells
NCCs: Neural crest cells
P7: postnatal day 7
NLBs: Neurosphere like bodies
*Dnmt3b*-KD: knockdown of *Dnmt3b* expression
shRNA: small hairpin RNA
KD: *Dnmt3b*-shRNA1
Non-Target: shRNA Non-Target Control group
C: uninfected group control
T-TBS: Tris-buffered salt solution with Tween
RIPA buffer: Radioimmunoprecipitation lysis buffer
SEM: Standard Error Mean
qRT-PCR: Quantitative Real-Time PCR
