# Supplementary material for: Dnmt3b knock-down in enteric precursors reveals a possible mechanism by which this de novo methyltransferase is involved in the enteric nervous system development and the onset of Hirschsprung disease
Source: Oncotarget. 2017 Nov 16;8(63):106443–53. doi: 10.18632/oncotarget.22473 (PMC5739746; doi:10.18632/oncotarget.22473)
Supplement: Supplementary file 1 [file oncotarget-08-106443-s001.pdf]

Dnmt3b knock-down in enteric precursors reveals a possible mechanism by which this *de novo* methyltransferase is involved in the enteric nervous system development and the onset of Hirschsprung disease

SUPPLEMENTARY MATERIALS

Supplementary Table 1: Positive expression of *Dnmt3b* in NLBs culture from mouse

	Dnmt3b Ct_m	beta-Actin Ct_m
Brain	27	18
NLBs 1	31	19
NLBs 2	22, 6	21, 3
NLBs 3	23	21, 1
NLBs 4	22, 4	21
NLBs 5	23	21,1
NLBs 6	25, 3	24, 5

Ct_m = Average of Ct from the triplicates.
Mouse brain tissue was used as positive control of *Dnmt3b* expression.
beta-Actin was used as positive control of qRT-PCR.

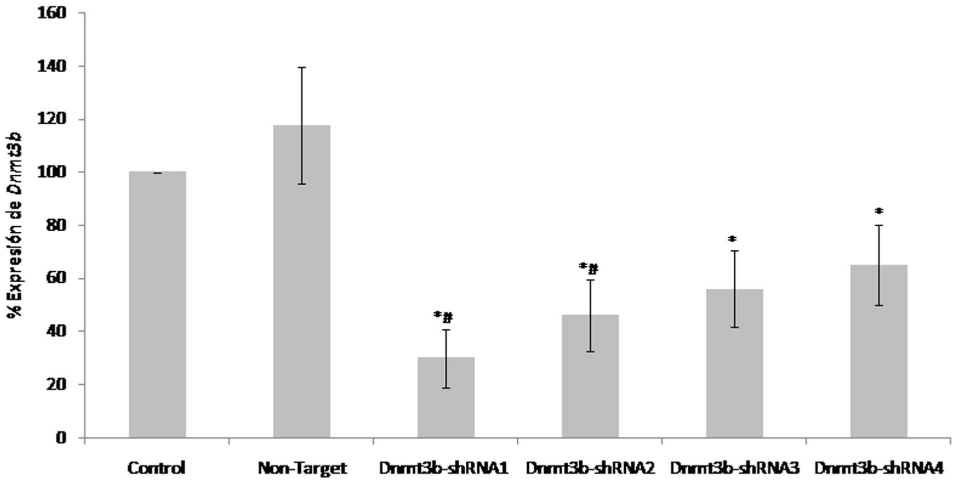

Supplementary Figure 1: Efficiency of each constructions of *Dnmt3b*-KD in NLBs cultures (*p* value *Dnmt3b*-shRNA1/C = 2.1794 E-05, *Dnmt3b*-shRNA2/C = 0.002, *Dnmt3b*-shRNA3/C = 0.009, *Dnmt3b*-shRNA 4/C = 0.02, *Dnmt3b*-shRNA1/Non-Target = 0.02, *Dnmt3b*-shRNA2/Non-Target = 0.01). Data are represented as mean ± SEM.

Supplementary Table 2: Primer sequences from each genes analyzed by qRT-PCR in the study

Gene	Forward primer sequence (5′-3′)	Reverse primer sequence (5′-3′)	Fragment size (pb)
p21	GTACTTCCTCTGCCCTGCTG	TCTGCGCTTGGAGTGATAGA	173
P21	CAGCAGAGGAAGACCATGTG	GGCGTTTGGAGTGGTAGAAA	153
Puma	GCCCAGCAGCACTTAGAGTC	TGTCGATGCTGCTCTTCTTG	191
Bax	TGCAGAGGATGATTGCTGAC	GATCAGCTCGGGCACTTTAG	173
Mdm2	TGCAAGCACCTCACAGATTC	ACACAATGTGCTGCTGCTTC	188
Casp6	GCAGAAGAACTCCTGCTCAAA	TTTGAACAAGCCAGTCAACG	156
Casp8	AACTGCGTTTCCTACCGAGA	GCATCTGCTTTCCCTTGTTC	169
P53	TGGCCATCTACAAGCAGTCA	GGTACAGTCAGAGCCAACCT	212

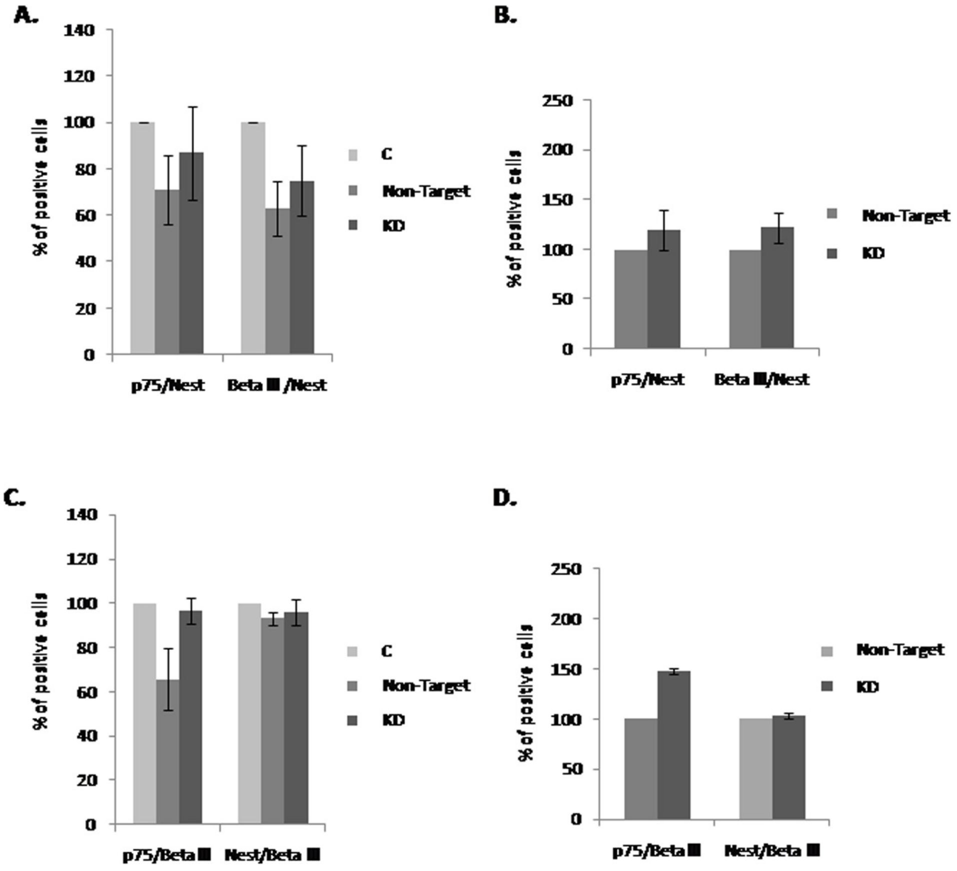

Supplementary Figure 2: (**A** and **B**) Graphs showing the effect of *Dnmt3b*-KD in the different culture conditions on the cells that express the markers combinations p75/Nest or β-III/Nest. (**C** and **D**) Graphs showing the effect of *Dnmt3b*-KD in the different culture conditions on the cells that express the markers combinations p75/β-III or Nest/β-III. Data are represented as mean ± SEM.
